# State Veterinary Bureaus

**Published:** 1882-01

**Authors:** 


State Veterinary Bureaus.—We have received from Dr. N. H. Paaren, State Veterinarian of Illinois, the first circular from his office. It contains the law enacted by the last Legislature under which a State Veterinary Bureau was created. It contains also a concise account of the leading symptoms in contagious pleuro-pneumonia, foot and mouth disease, malignant anthrax, and Texas or splenic fever. The facts thus given are such as ought to be familiar to every owner and dealer in stock. Their distribution cannot but have a beneficial effect.

The State Veterinarian, besides thus distributing information, has other important duties. These consist in keeping a careful watch over the herds and over stock-yards; also in examining cattle that are imported from the East. Quite recently the Governor of Illinois issued a proclamation forbidding the bringing of cattle into the State from regions affected with pleuro-pneumonia, unless these cattle had a certificate of health properly signed by a duly authorized veterinary inspector. Whether such a prohibition will or can be carried out is at present doubtful. But, at any rate, the object is a good one, and will lead to an agitation of the question of stamping out pleuro-pneumonia, which cannot but be useful.

It has already been shown that the State Veterinary Department of Illinois has a wide field for work. A great many will watch it with interest, for if it succeeds in justifying its existence, as it no doubt can, a precedent which other States must follow will be established.

